# Enhancing Degradation Resistance of Biomedical Mg-6Zn-0.5Zr Alloy by the Incorporation of Nanodiamond

**DOI:** 10.3390/ma15196707

**Published:** 2022-09-27

**Authors:** Long Liu, Shun He, Zhiming Guo, Jian Li, Mingchun Zhao, Yiping Wu

**Affiliations:** 1 Department of Mechanical and Electrical Engineering, Changsha University, Changsha 410003, China; 2 School of Materials Science and Engineering, Central South University, Changsha 410083, China

**Keywords:** selective laser melting, Mg alloys, nanodiamond, apatite layer, degradation resistance

## Abstract

The Mg-6Zn-0.5Zr (ZK60) alloy has attracted extensive attention as one of the hopeful biomedical material candidates for bone implant applications on account of its unique degradability, favorable biocompatibility as well as mechanical compatibility. Nevertheless, the rapid degradation rate in the biological environment is the major hurdle for its clinical application in the field of bone implants. In this study, nanodiamond (ND) was incorporated into ZK60 alloy via selective laser melting technology to enhance its degradation resistance. The results showed that compared with selective laser-melted ZK60 (SLMed ZK60), the selective laser-melted ZK60 with 6 wt.% ND (SLMed ZK60−6ND) possessed the better degradation resistance with the lower degradation rate of 0.5 ± 0.1 mm/year. The enhancement of the degradation resistance was attributed to the fact that ND could promote the deposition of apatite and build up a dense and insoluble protective layer through the dissociation of the carboxyl groups on the ND surface, which could effectively hinder the further degradation of the Mg matrix. Meanwhile, the compressive strength and hardness were improved mainly due to grain refinement strengthening and ND dispersion strengthening. In addition, the SLMed ZK60−6ND possessed good cytocompatibility. These results suggested that the SLMed ZK60−6ND, with enhanced degradation resistance, improved mechanical properties, and good cytocompatibility, was an excellent biomedical material candidate for bone implant applications.

## 1. Introduction

In the advanced field of medical sciences, magnesium (Mg) and its alloys have achieved considerable attention of researchers and emerged as the potential candidates for a new generation of degradable bone implant materials, owing to their inherent degradability, good biocompatibility and suitable mechanical properties [1,2,3,4,5]. Mg alloys have a low electrode potential (−2.37 V) as well as strong chemical activity. They can be gradually degraded and absorbed after implantation in the body, avoiding the implant removal operation [6]. Meanwhile, Mg is an essential element to the human body and beneficial to the growth of new bone tissue [7]. In addition, their density and elastic modulus (density: 1.74–2.04 g/cm^−3^, elastic modulus: 41–45 GPa) are close to those of human bone (density: 1.8–2.1 g/cm^−3^, elastic modulus: 10–30 GPa), which can avoid the stress shielding problem [8]. Currently, numerous types of Mg alloys have been studied as biomedical materials for bone implant applications, such as Mg-3Al-1Zn (AZ31) alloy, Mg-6Al-1Zn (AZ61) alloy, Mg-4.32Y-2.83Nd-0.41Zr (WE43) alloy and Mg-6Zn-0.5Zr (ZK60) alloy [9,10,11,12,13,14]. Among them, ZK60 alloy has excellent mechanical and biological properties. Nevertheless, the degradation rate of ZK60 is too rapid to satisfy the needs of bone repair [15,16]. The rapid degradation may lead to hydrogen gas accumulation, local alkalization and loss of mechanical integrity prior to tissue healing, which limit the clinical application of ZK60 as a bone implant material.

One cause for the rapid degradation of ZK60 alloy in physiological environment is that the degradation product layer on ZK60 alloy is a porous and soluble magnesium hydroxide layer, which cannot play a role in preventing further degradation of the Mg matrix [17,18]. As we all know, for metals such as aluminum, titanium, chromium and stainless steel, the high corrosion resistance is put down to the formation of a dense and insoluble corrosion product layer on the alloy surface, thereby preventing the ongoing reaction of an aggressive medium with the alloy matrix [19,20]. Inspired by this idea, two strategies have been put forward to enhance the degradation resistance of ZK60 alloy. The first strategy was to alloy it with other elements which could facilitate formation of a protective film on its surface during degradation [21,22]. The second strategy was to coat the surface with a high-degradation-resistance protective layer to improve the corrosion resistance of ZK60 alloy [23,24]. However, both strategies have their own limitations. For the first strategy, most of the alloying elements are nobler than Mg and have limited solubility in Mg. They mostly precipitate as intermetallic compounds which act as local cathodes causing the micro galvanic corrosion to accelerate the degradation of ZK60 alloy. For the second strategy, the coating layer only provides a solution for retarding the initial corrosion, while rapid corrosion can occur once the coating layer ruptures. Thus, facilitating a protective layer sustainably forming on the alloy surface without causing micro galvanic corrosion may be one promising strategy for enhancing the bulk degradation resistance of Mg.

In recent years, nanodiamond (ND) has received increased attention in bone tissue engineering, due to its outstanding properties of high hardness, low coefficient of friction, high chemical stability and insulating property, good biocompatibility and easily modifiable surfaces [25,26,27,28]. ND is a class of carbon material with a particle size less than 100 nm and with abundant oxygen-containing functional groups on its surface such as hydroxyl and carbonyl. It has the dual characteristics of diamond and nanomaterial. On the one hand, as a diamond material, ND possesses high hardness, high chemical stability and an electrical insulating nature. On the other hand, as a nanomaterial, ND has a large specific surface area and high surface activity. Furthermore, previous studies revealed that ND could promote apatite deposition in the physiological environment duo to the hydrolysis of functional groups on the ND surface [29,30]. These characteristics motivated us naturally to hypothesize that an apatite layer might be formed on the surface of ZK60 alloy, and micro galvanic corrosion would not occur if ND was incorporated into it. Additionally, the apatite layer on the surface can offer more effective protection as compared with a magnesium hydrate layer. Thus, incorporating ND into ZK60 alloy may be beneficial for enhancing its degradation resistance. Therefore, thus far, there are only a few studies about the influence of ND on the mechanical capacities and thermal properties of Mg alloys, while there is no report on the influence of ND on their biodegradation behavior [31,32].

In this study, ND was incorporated into ZK60 alloy to enhance its degradation resistance via the selective laser melting (SLM) technique. The SLM technique is a typical rapid solidification technique with a cooling rate over 10^5^ K/s, which is able to inhibit grain growth and reduce composition segregation. The fine grain and homogeneous microstructure are believed to be beneficial in enhancing mechanical properties and degradation resistance of Mg alloys. The effects of ND on the microstructure evolution, degradation behavior, mechanical properties and cytocompatibility of ZK60 alloy were investigated intensively.

## 2. Experiment

### 2.1. Material and Sample Fabrication

**Figure 1:**
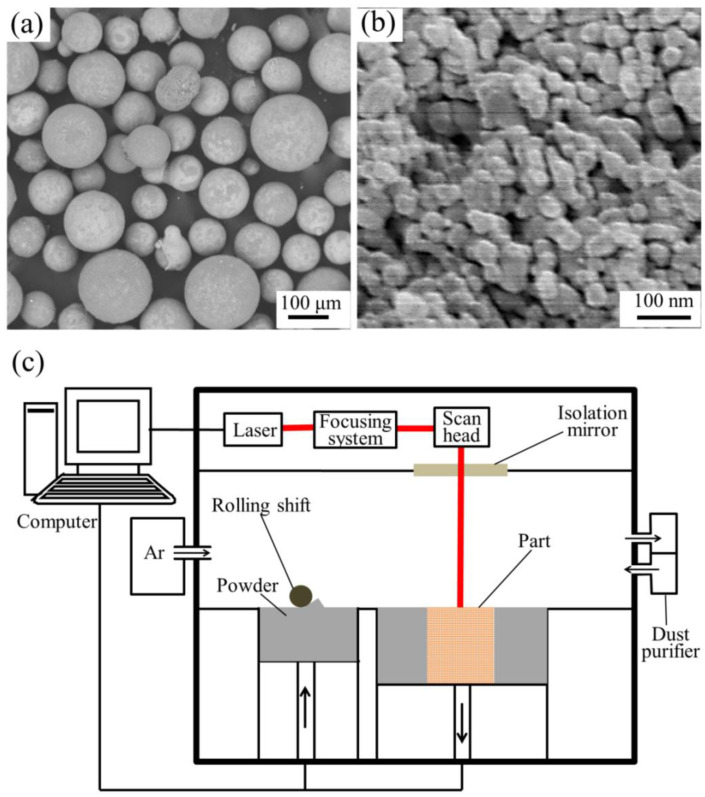
Morphology of (**a**) ZK60 alloy powder and (**b**) ND powder; (**c**) the schematic diagram of SLM process.

The gas-atomized spherical ZK60 alloy powder with the average particle size of 100 μm was purchased from Tangshan Weihao Magnesium Powder Co. Ltd. (Figure 1a). The detonation nanodiamond (ND) with an average particle size 40 nm was purchased from Shanghai Naiou Nano technology Co., Ltd. (Figure 1b). The ZK60−xND (where x = 0, 3, 6, 9 wt.%, denoted as ZK60, ZK60−3ND, ZK60−6ND and ZK60−9ND, respectively) mixed powder was prepared as follows: first, the ND was dispersed in alcohol solution by ultrasonic dispersion for 20 min in order to obtain a ND suspension. Subsequently, the ZK60 powder was added to the ND suspension, followed by being mechanically stirred. Then, the suspension was filtered and dried in a vacuum oven, at room temperature, to completely remove ethanol.

A homemade selective laser melting (SLM) piece of equipment was used to fabricate ZK60−xND. The schematic diagram of SLM process was shown in Figure 1c. First, a thin layer of original powder was paved on the substrate by a rolling shift. Then, the laser scanning path was controlled by the computer and laser completely melted the paved powder. After each layer was melted, the forming cylinder dropped the height of one layer, and then powder was repaved and completely melted according to the preset path. The above steps were repeated until target samples were prepared. The optimal processing parameters for the selective laser-melted (SLMed) ZK60−xND were as follows: laser power 80 W, spot diameter 200 μm, scan space 80 μm and scan speed 50 mm/s.

### 2.2. Microstructural and Mechanical Characterization

The metallographic structure of SLMed ZK60−xND was characterized by optical microscope. Before the optical microscope observation, the samples were polished and then etched with 4 vol.% nitric acid ethanol solution for 10 s. The phase composition and distribution were studied by scanning electron microscopy equipped with energy-dispersive spectroscopy (SEM-EDS, JCM 6000, JEOL Ltd., Tokyo, Japan) and X-ray diffraction (XRD, D8 Advance, Bruker Inc., Karlsruhe, Germany). The Raman spectra were recorded in the region from 800 cm^−1^ to 2000 cm^−1^ by a focused Raman spectrometer (LabRAM HR800, Horiba Jobin-Yvon, Villeneuve d’Ascq, France). The relative density of the SLMed ZK60−xND was measured by the Archimedes method. The relative density (ρ_relative_) was calculated using the formula ρ_relative_ (%) = ρ/ρ_theoretical_ × 100%, where ρ is the actual density of the sample and ρ_theoretical_ is theoretical density for ZK60 (1.83 g/cm^3^). Vickers hardness and compression tests of the samples were measured on the microhardness tester using a load of 2.942 N for 15 s and electronic universal testing machine using a crosshead speed of 0.2 mm/min, respectively.

### 2.3. Degradability Characterization

Potentiodynamic polarization tests were performed in simulated body fluid (SBF) at 37 ± 0.5 °C using an electrochemical workstation. A typical three-electrode system was applied, where the sample with an exposed surface area of 1 cm^2^ was the working electrode, the platinum plate was the counter electrode, and the reference electrode was a saturated calomel electrode. The potential was scanned from −1.8 V to −1.2 V at a scan rate of 0.5 mV/s. Immersion tests were executed in SBF at 37 ± 0.5 °C for 240 h. Before the immersion test, all samples were mechanically ground by 1200 grit silicon carbide abrasives and polished with 0.5 μm diamond brightener. The pH value of the immersion solution was measured by a digital pH meter for every 12 h. The volume of evolved hydrogen gas (H_2_) was recorded during the immersing process. After immersing for 240 h, the samples were taken out from the SBF and rinsed with distilled water three times, then dried by the blower at room temperature. The surface morphology was characterized by SEM-EDS. For the weight loss test, the initial weight of the specimen was recorded before immersing. After immersing for 240 h, the samples were cleaned with chromic acid solution to remove the degradation products before mass loss measure. The biodegradation rate Pw (mm/y) was estimated from the weight loss rate W (mg/cm^−2^/d^−1^) using the formula Pw = 2.1 W.

### 2.4. Cytocompatibility Characterization

The cytocompatibility of the samples was assessed using a human osteoblast-like cell line MG63. Dulbecco’s modified Eagle’s medium (DMEM) supplemented with 10% heat-inactivated fetal bovine serum were used as culture medium. The SLMed samples were soaked in Dulbecco’s modified Eagle’s medium with a surface-area-to-extraction-volume ratio of 1.25 cm^2^/mL^−1^ for 5 days, and then the extracts were collected. For fluorescent staining assays, MG-63 cells were cultured in a 24-well culture plate with DMEM for 24 h. Then, the DMEM medium were removed and replaced by the prepared extracts. After incubating for 1, 3 and 5 days, respectively, the cells were gently rinsed with phosphate-buffered saline, subsequently stained using Calcein-AM and Ethidium homodimer-1 reagents for 15 min, and finally visualized by a fluorescence microscope.

For Cell Counting Kit-8 (CCK-8) assays, the MG63 cells at a density of 5 × 10^4^ cells per well were seeded in the culture plate for 24 h to allow attachment and the medium was removed subsequently. Then, 100 mL of the prepared extracts was added into the well. After incubating for 1, 3, and 5 days, respectively, 10 μL of CCK-8 reagent was added to each well, and the cells were incubated further for another 1 h, at 37 °C. The absorbance was measured at a wavelength of 450 nm using a microplate spectrophotometer. For cell adhesion morphology analysis, the samples were settled in 6-well plates and 1 mL MG-63 cells suspensions were added in each well. After culturing for 5 days, cells were washed with PBS and then fixed with 4% paraformaldehyde, then sequentially dehydrated using a series of ethanol solutions. The morphologies of MG63 cells on the samples were observed by SEM after being sputter-coated with gold.

### 2.5. Statistical Analysis

The data obtained in this study were expressed as mean ± standard error. In order to evaluate the significance of the differences between groups, the Student *t*-test was conducted. It was considered to be significantly different if the *p*-value was less than 0.05.

## 3. Results and Discussion

### 3.1. Microstructure

**Figure 2:**
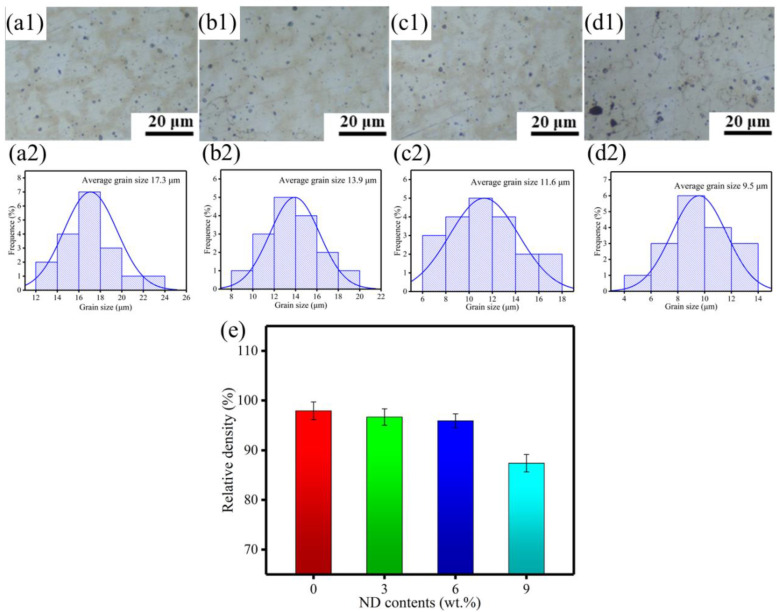
Metallographic structure and corresponding grain size distribution of ZK60−xND: (**a1**,**a2**) ZK60, (**b1**,**b2**) ZK60−3ND, (**c1**,**c2**) ZK60−6ND, (**d1**,**d2**) ZK60−9ND, respectively; (**e**) relative density of SLMed ZK60−xND.

The metallographic structure and corresponding grain size distribution of selective laser-melted (SLMed) ZK60−xND are shown in Figure 2(a1–d1),(a2–d2), respectively. It can be seen that all SLMed ZK60−xND consist of equiaxed grains. Their average grain sizes were further measured by the linear intercept method. With the increase in ND content in ZK60 alloy, the average grain size rapidly decreases from 17.3 μm for SLMed ZK60 to 11.6 μm for SLMed ZK60−6ND, and then slightly decreases to 9.5 μm for the ZK60−9ND. The results indicate that the ND introduction can refine the grain size, and the ZK60−6ND has the smallest grain size. The grain refinement mechanism is as follows: (1) during the solidification process, the enrichment of ND at the front of the solid/liquid interface can induce constitution undercooling at the solidification interface front, thus facilitating nucleation and refining the grains; (2) in addition, the ND precipitates at the grain boundary during solidification also can hinder grain growth.

The densification is a critical factor influencing the degradation and mechanical properties of SLMed Mg alloys. Therefore, the relative density of the SLMed ZK60−xND was determined by using the Archimedes method, and the results are shown in Figure 2e. The relative density of SLMed ZK60 is 97.9 ± 1.8% (actual density 1.79 g/cm^3^). After incorporation of ND, ZK60−3ND and ZK60−6ND still maintain the high-level relative densities of 96.6 ± 1.6% (actual density 1.77 g/cm^3^) and 95.9 ± 1.4% (actual density 1.75 g/cm^3^), respectively. The results show that highly dense ZK60−ND can be fabricated by the SLM process. However, as the ND content increases to 9 wt.%, the relative density decreases significantly to 87.4 ± 1.7% (actual density 1.60 g/cm^3^), which is significantly lower than that of SLMed ZK60. It is indicated that excessive ND will deteriorate the densification behavior of SLMed ZK60−xND. Generally, during the SLM process of ZK60−xND, the ZK60 alloy powder is completely melted into the liquid phase, while the higher melting point ND remains in the solid phase. Excessive solid ND tends to aggregate together during the solidification process. The liquid metal cannot wet and fill the interstice of the agglomerated ND, thereby reducing the densification rate of the ZK60−xND.

**Figure 3:**
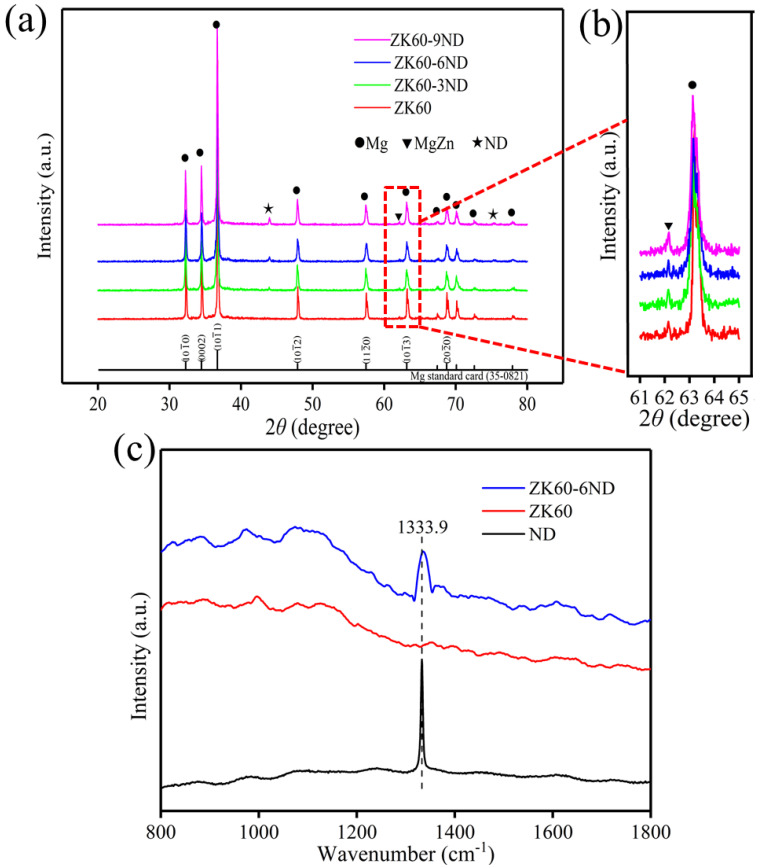
(**a**) The XRD pattern of SLMed ZK60−xND; (**b**) the enlarged area of the XRD pattern (2*θ* = 61–65°); (**c**) Raman spectrum of SLMed ZK60, SLMed ZK60−6ND as well as the original ND.

The XRD patterns of the SLMed ZK60−xND are shown in Figure 3a,b. It is shown that SLMed ZK60 is constituted of α-Mg and Mg-Zn phase. With the incorporation of ND, the diffraction peaks of ND appear in ZK60−6ND and ZK60−9ND, which indicates that ND is successfully incorporated into ZK60 during the laser melting process. To further determine the status of ND in SLMed ZK60−xND, the Raman spectra were recorded for SLMed ZK60 and SLMed ZK60−6ND, using the original ND as the control. The original ND exhibits one sharp peak at 1333.9 cm^−1^, which corresponded to the sp^3^ bond of diamond [33]. No Raman spectrum is observed in SLMed ZK60, while a Raman spectrum peak near 1333.9 cm^−1^ is observed for the SLMed ZK60−6ND, which indicates that the structure of the ND does not change in the SLM process. The XRD and Raman results show that there are no changes to the phase and structure of ND during the SLM process due to the rapid melting/solidification characteristic of SLM.

**Figure 4:**
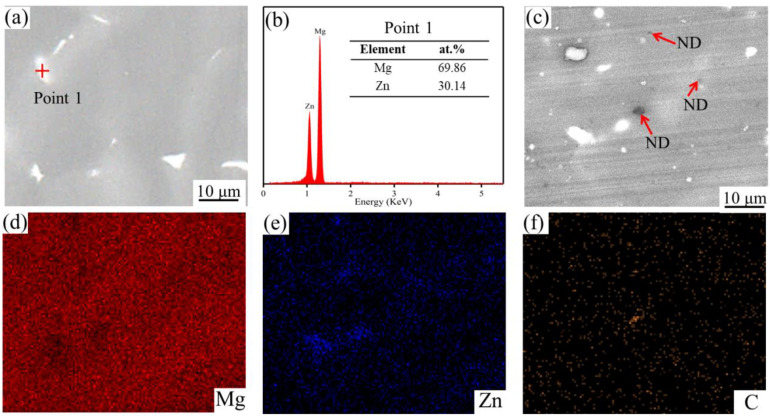
(**a**) SEM image of SLMed ZK60 and (**b**) corresponding EDS of point 1; (**c**) SEM image of SLMed ZK60−6ND and its corresponding EDS map of (**d**) Mg, (**e**) Zn, and (**f**) C element.

The composition and distribution of SLMed ZK60 and SLMed ZK60−6ND were analyzed by SEM-EDS, as shown in Figure 4. The SLMed ZK60 is composed of a gray α-Mg matrix and a white secondary phase (Figure 4a). The white secondary phase in ZK60 is sparsely distributed along the grain boundaries and has an island-like structure. The EDS results show that the white island-like second phase (point 1 in Figure 4a) consists of Mg (69.86 at.%) and Zn (30.14 at.%). Combined with the XRD results, it is indicated that the white island-like second phase is the Mg-Zn phase, while for the ZK60−6ND, the black particles (as pointed by red arrows) are observed (Figure 4c). The EDS map results show that the Mg element is homogeneous in structure, the Zn element is in the white particles at the grain boundaries, and these black particles are rich in C element distributed throughout the structure, indicating that the matrix is composed of the Mg element, and the ND is uniformly distributed in the matrix (Figure 4d,e). The uniform distribution of the ND in the Mg matrix is mainly attributable to the rapid melting and solidification hindering the agglomeration of ND during the SLM process.

### 3.2. Degradation Properties

**Figure 5:**
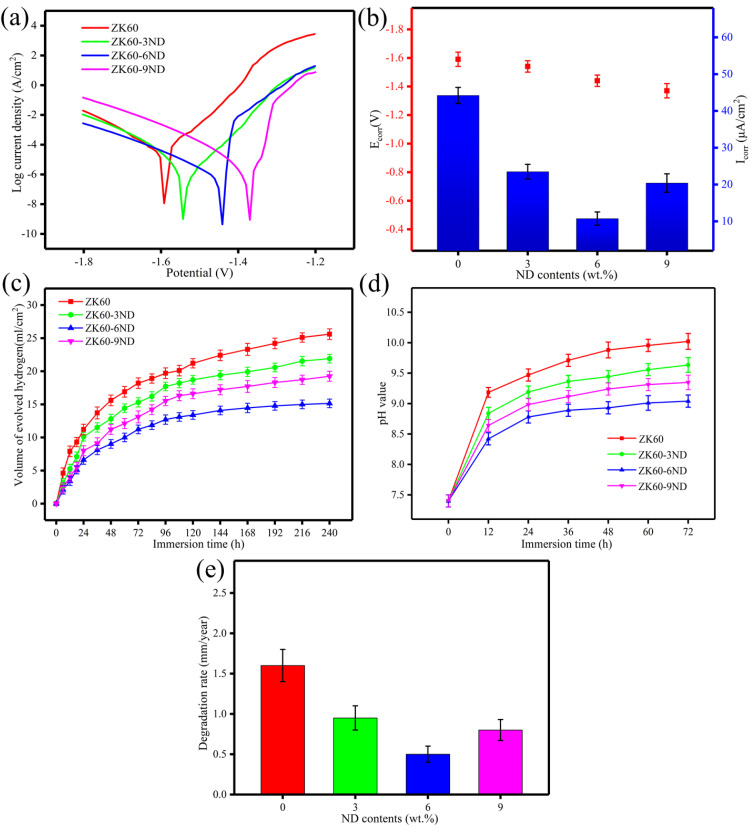
(**a**) Polarization curves of SLMd ZK60−xND and (**b**) corresponding corrosion potential (*E*_corr_) and corrosion current density (*I*_corr_) obtained from polarization curves; (**c**) the variations of hydrogen evolution volume of SLMed ZK60−xND immersed in SBF; (**d**) variation of pH value; (**e**) degradation rate based on weight loss test.

The polarization curves of the SLMed ZK60−xND in simulated body fluid (SBF) are shown in Figure 5a. The corresponding corrosion potential (*E*_corr_) and corrosion current density (*I*_corr_) are shown in Figure 5b. The *E*_corr_ of the SLMed ZK60 is relatively low at −1.59 ± 0.05 V. With the content of ND increasing, the *E*_corr_ gradually shifts to the positive direction. The *E*_corr_ of the SLMed ZK60−6ND and ZK60−9ND are −1.44 ± 0.04 V and −1.37 ± 0.05 V, respectively. Typically, the higher the corrosion potential is, the more difficult the corrosion process occurs. The incorporation of ND into ZK60 increases its corrosion potential, which means that the degradation resistance of ZK60 is enhanced by the incorporation of ND. The *I*_corr_ is 44.21 ± 2.32 μA/cm^2^ (SLMed ZK60), 23.47 ± 2.13 μA/cm^2^ (SLMed ZK60−3ND), 10.73 ± 1.84 μA/cm^2^ (SLMed ZK60−6ND) and 20.39 ± 2.51 (SLMed ZK60−9ND). The SLMed ZK60−6ND possesses the lowest *I*_corr_. Generally, the smaller the *I*_corr_ value, the higher the degradation resistance of the alloy. Therefore, the polarization curve results mean that the SLMed ZK60−6ND has the highest degradation resistance.

The variations of hydrogen (H_2_) evolution volume after soaking ZK60-xND in SBF for different periods are shown in Figure 5c. For all samples, the H_2_ evolution volume increases rapidly in the first 48 h, and then increases slowly and finally stabilizes. In the initial stage of immersion, the Mg matrix contacts with SBF directly and rapidly degrades. As the degradation progressed, a large amount of degradation products cover the surface, which in turn acts as a protective layer to prevent the degradation, and therefore, the H_2_ evolution volume increases slowly after a period of time. After immersing for 240 h, the volume of H_2_ released by the SLMed ZK60 is 25.6 mL/cm^2^. Compared with the SLMed ZK60, the SLMed ZK60−3ND and the SLMed ZK60−6ND release the lower volume of hydrogen 21.9 mL/cm^2^ and 15.1 mL/cm^2^, respectively (Figure 5c). With the further increase in ND content, the hydrogen evolution volume of the SLMed ZK60−9ND increases to 19.2 mL/cm^2^. Furthermore, the pH variation during immersion of SLMed ZK60−xND in SBF is shown in Figure 5d. The pH values for all SLMed ZK60−xND increase rapidly in the initial 12 h, while increasing slowly with increasing immersion time. The pH value of immersion solution corresponding to SLMed ZK60, SLMed ZK60−3ND, SLMed ZK60−6ND and SLMed ZK60−9ND reaches 10.02 ± 0.13, 9.63 ± 0.12, 9.04 ± 0.10 and 9.35 ± 0.12 at the end of the immersion tests, respectively.

In addition, the degradation rates of the samples are quantified by the weight loss test as shown in Figure 5e. The degradation rate is 1.6 mm/year for SLMed ZK60, 0.95 mm/year for SLMed ZK60−3ND, 0.5 mm/year for SLMed ZK60−6ND, and 0.8 mm/year for SLMed ZK60−9ND. These results show that the SLMed ZK60−6ND has the lowest degradation rate, indicating that proper ND incorporation can enhance the degradation resistance of ZK60.

**Figure 6:**
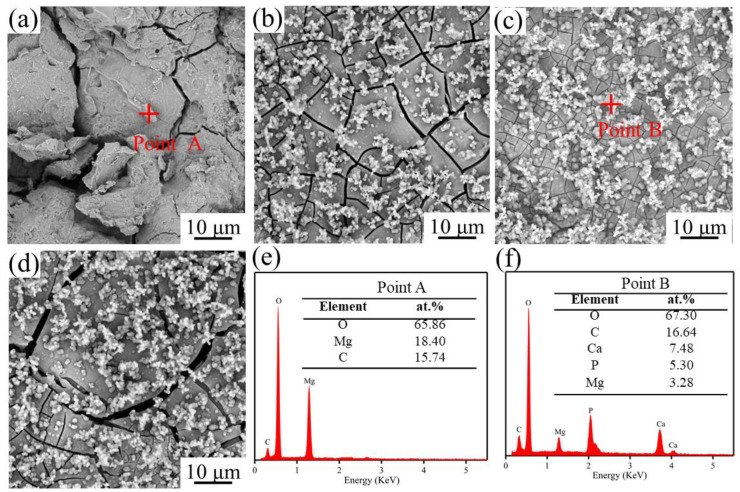
The morphology of the degradation product on the SLMed ZK60−xND after immersion in SBF: (**a**) SLMed ZK60; (**b**) SLMed ZK60−3ND; (**c**) SLMed ZK60−6ND; (**d**) SLMed ZK60−9ND; (**e**) EDS result of the point A in SLMed ZK60 and (**f**) point B in SLMed ZK60−6ND.

To further investigate the influence of ND on the degradation behavior of SLMed ZK60−xND, the degraded surfaces of the SLMed ZK60−xND in the SBF were investigated (Figure 6). After immersion for 240 h, the SLMed ZK60 exhibits loose surface features with many corrosion cracks (Figure 6a), while the SLMed ZK60−6ND exhibits relatively dense surface features, and a lot of spherical precipitates appear on the degradation surface (Figure 6c). For SLMed ZK60−9ND, some big cracks appear in localized areas of the surface, and more spherical precipitates are observed on the surface compared to the SLMed ZK60−6ND. This phenomenon shows that the least amount of degradation products forms on the SLMed ZK60−6ND.

EDS analysis was used to further analyse the composition of degradation product. The results show that the degradation product on SLMed ZK60 was mainly constituted of Mg and O (Figure 6e), manifesting that the degradation product on SLMed ZK60 is magnesium hydroxide (Mg(OH)_2_), while a large amount of calcium (Ca) and phosphorus (P) are detected in the degradation product of ZK60−6ND (Figure 6f), and the Ca/P ratio is about 1.5 which is close to that of apatite (1.67) [34]. It is indicated that the spherical particles formed on the surface of ZK60−6ND are apatite. It is reasonable to conclude that the incorporation of ND can promote the formation of apatite on the ZK60−xND surface in the physiological environment.

**Figure 7:**
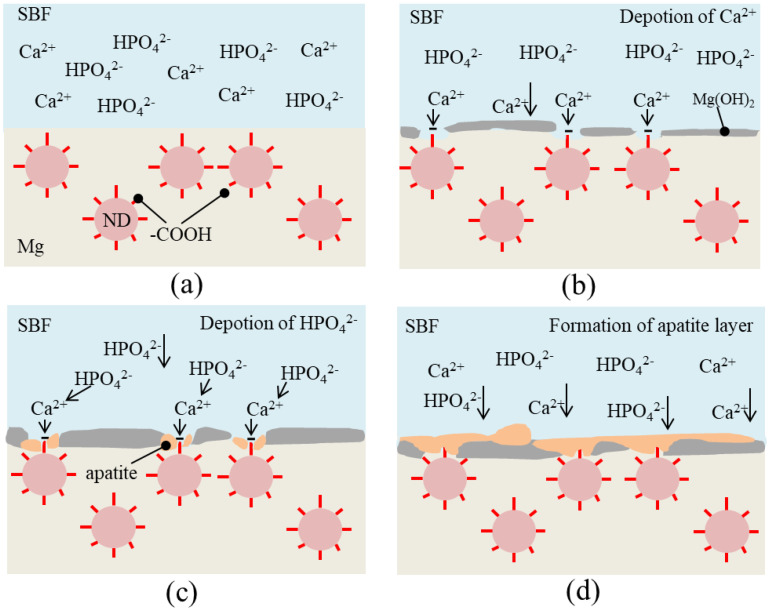
Schematic diagram of the mechanism of ND enhancing the degradation resistance of SLMed ZK60−xND: (**a**) the initial stage; (**b**) deposition of Ca^2+^; (**c**) deposition of HPO_4_^2−^; (**d**) formation of apatite layer.

The schematic diagram of the mechanism of ND enhancing the degradation resistance of SLMed ZK60−xND is shown in Figure 7. The degradation of SLMed ZK60−xND in SBF usually occurs through electrochemical reaction (Figure 7a). The α-Mg matrix reacts with the corrosive medium to generate Mg(OH)_2_ and H_2_. Then, as the degradation process proceeded, the NDs in the SLMed ZK60−xND are exposed to SBF. The carboxyl groups (–COOH) on the ND surface will dissociate in SBF, which make the SLMed ZK60−xND surface negatively charged and attract Ca^2+^ in the SBF to the surface through electrostatic adsorption (Figure 7b). The deposition of Ca^2+^ is positively charged, and they in turn interact with the negatively charged hydrogen phosphate (HPO_4_^2−^) in SBF. Such a migration of Ca^2+^ and HPO_4_^2−^ to the SLMed ZK60−xND surface facilitates the formation of apatite clusters (Figure 7c). As a result, the apatite clusters then gradually grow to form a dense and insoluble apatite layer on the surface, which acts as a protective layer to protect the Mg matrix from the intrusion of the SBF (Figure 7d). Therefore, the SLMed ZK60−xND obtains an enhanced degradation resistance.

### 3.3. Mechanical Properties

**Figure 8:**
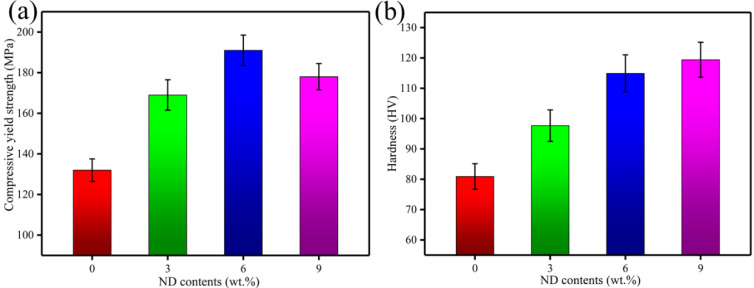
Compression strength (**a**) and hardness (**b**) of SLMed ZK60-xND.

The compressive strength and hardness of SLMed ZK60−xND are shown in Figure 8. With increasing ND content, the compressive strength increases first from 132 ± 5.5 MPa for SLMed ZK60 to 191 ± 7.5 MPa for SLMed ZK60−6ND, while it decreases with further increasing ND content. The enhancement in compressive strength is put down to grain refinement and the uniformly dispersed ND, which effectively limited the movement of dislocations. The grain size refinement results in an enhancement in the fraction of the grain boundary. Grain boundaries act as a barrier to dislocation motion, one of the main factors of strengthening. Furthermore, the uneven incorporation of ND particles into the Mg matrix can function as barriers, obstructing dislocation motion. However, excessive ND agglomeration leads to the accumulation of stress at the interface of ND and Mg matrix during deformation, leading to a decrease in compressive strength. Furthermore, the hardness of SLMed ZK60−xND was investigated. With increasing ND content, the hardness of SLMed ZK60−xND increases from 80.9 ± 4.2 HV for SLMed ZK60 to 119.4 ± 5.7 HV for SLMed ZK60−9ND. The hardness increases continuously with the increase in ND content, which is attributed to the presence of the harder ND. The hard ND randomly dispersing in the matrix can prevent dislocation movement during deformation, resulting in a significant hardening effect.

### 3.4. Cytocompatibility

**Figure 9:**
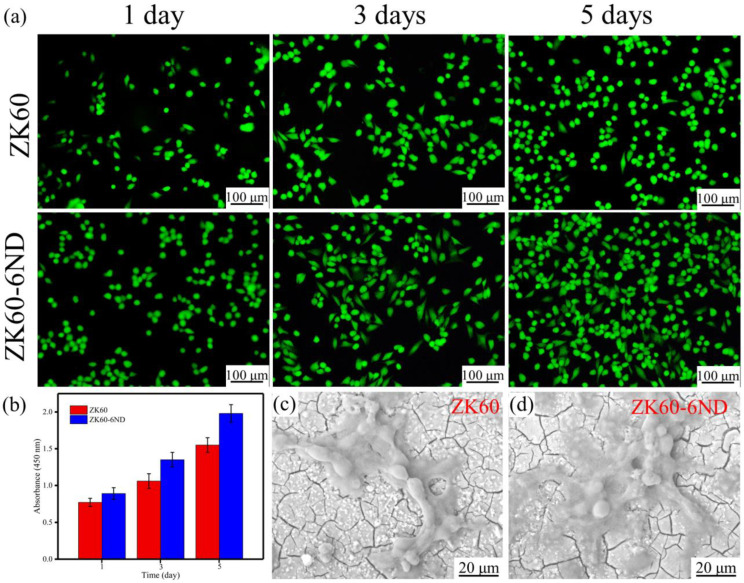
(**a**) Fluorescent images of MG-63 cells cultured in extracts for 1, 3, and 5 days; (**b**) CCK-8 results in extracts for 1, 3, and 5 days; morphologies of MG-63 cells cultured on (**c**) SLMed ZK60 and (**d**) SLMed ZK60-6ND.

As a bone implant material, the cytocompatibility of SLMed ZK60−xND was evaluated. The SLMed ZK60−6ND with the best comprehensive properties was selected as the representative, and the SLMed ZK60 was used as the control. MG-63 cells were co-cultured with the SLMed ZK60−6ND and ZK60 extract for 1, 3 and 5 days, respectively. Then, they were investigated by fluorescent staining (Figure 9a). The results show that cells in both SLMed ZK60−6ND and ZK60 exhibit a marked spindle shape, and no dead cells are detected, indicating normal growth behavior. In addition, when the incubation time increases to 5 days, the quantity of cells also increases. Additionally, for the same cultured time, the viable cells for the SLMed ZK60−6ND are more than that for the SLMed ZK60. The cell fluorescence results indicate that the SLMed ZK60−6ND is more conducive to cell growth than SLMed ZK60.

CCK-8 assays were also conducted to the quantitative analysis of cell viability (Figure 9b). The optical density of all samples increases when the incubation time increases from 1 day to 5 days, indicating the proliferation of MG-63 cells. The higher optical density is observed in SLMed ZK60−6ND compared to SLMed ZK60. Furthermore, to observe the morphologies of MG-63 cells culturing with the samples, it is found that MG-63 cells adhere and expand on the samples and the cells on SLMed ZK60−6ND display larger cell spreading area and more pseudopodia compared with those on SLMed ZK60 (Figure 9c,d). The above results indicate that the SLMed ZK60−6ND possesses the better cytocompatibility than SLMed ZK60. It is attributable to the slower degradation rate of SLMed ZK60−6ND, which significantly reduces the release of Mg^2+^ and OH^−^ during the degradation, resulting in a more suitable pH and ions concentration for cell survival than SLMed ZK60.

## 4. Conclusions

In the present research, nanodiamond (ND) was incorporated into ZK60 alloy to enhance the degradation resistance via selective laser melting (SLM) technique. It was found that the selective laser-melted (SLMed) ZK60−6ND (degradation rate 0.5 mm/y) exhibited much higher degradation resistance than SLMed ZK60 (degradation rate 1.6 mm/y). The reason for the enhanced degradation resistance was attributed to the fact that ND could promote the deposition of apatite and build up a dense and insoluble protective layer which could effectively hinder the further degradation of the Mg matrix. Furthermore, the incorporation of ND could significantly enhance the mechanical properties of ZK60 through grain refinement and ND dispersion strengthening. Meanwhile, the SLMed ZK60−6ND exhibited better cytocompatibility than the SLMed ZK60. Thus, it is proposed that the SLMed ZK60−6ND is a promising bone implant material for biodegradable implant application.

## Data Availability

Not applicable.
